# Strategies for Implementing Research Inspired Teaching in Higher Education for Midwifery and Nursing: A Qualitative Study Introducing the 4M Model

**DOI:** 10.1177/23333936251369833

**Published:** 2025-11-12

**Authors:** Elina Leiviska, Sally Pezaro, Luca Morini, Alun DeWinter, Rosie Kneafsey

**Affiliations:** 1 Coventry University, United Kingdom; 2 Loughborough University, United Kingdom

**Keywords:** midwifery and nursing education, research inspired teaching, evidence-based practice, qualitative research, United Kingdom

## Abstract

Integrating research and evidence into teaching promotes critical thinking, problem solving and innovation, but challenges remain in identifying the most effective methods. The Research Inspired Online/Offline Teaching (RIOT) framework presents a solution for midwifery and nursing education, but its applicability and feasibility must first be tested. This study investigated factors influencing the implementation of the RIOT framework and proposed solutions for overcoming barriers. Semi-structured interviews and qualitative surveys were conducted with academic staff, and educational managers and leaders in a university in the United Kingdom between February and June 2024. Twenty-four interviews and six qualitative survey responses were analysed using framework and thematic analysis. Framework analysis identified barriers and solutions at five levels: individual, interpersonal, organisational, institutional and societal. Thematic analysis formed four overarching themes: (1) understanding the relevance of research, (2) knowledge, skills, tools and confidence to apply research into teaching, (3) establishing a collaborative research culture and (4) overcoming challenges impacting the ability to maintain teaching excellence. The 4M Model for Promoting Research Inspired Teaching in Midwifery and Nursing Education was developed from the findings to provide a foundation for facilitating research integration via the RIOT framework. The 4M model emphasises the necessity for collaboration, optimisation of research impact, cultivation of a collaborative research culture, and maintenance of teaching excellence.

## Introduction

Teaching and research activities in Higher Education Institutions (HEIs) represent two of the most significant aspects of their operations. The relationship between these two core functions is a topic of considerable interest within the academic community (Dandridge, 2023; Hays et al., 2020; McKinley et al., 2021; Uaciquete & Valcke, 2022). Integrating research into the teaching curriculum has positive effects on students’ subject knowledge, critical thinking, problem-solving and professional decision-making abilities (Burke-Smalley et al., 2017; McCormick et al., 2015; Pérez-Perdomo & Zabalegui, 2024). Research skills are essential for discovery, knowledge transfer and innovation, and for producing evidence for professional practice (Weston et al., 2017) as well as to generate new knowledge or validate existing knowledge based on theory (Conner, 2014). Despite these benefits, gaps remain in identifying the best ways to integrate research into teaching (Bala et al., 2021; Georgiou et al., 2023; Hirsh et al., 2022). It is therefore crucial to develop teaching strategies and models that enhance this relationship in HEIs.

The Research Inspired Online/Offline Teaching (RIOT) framework (Pezaro et al., 2022) was co-created to bridge the gap between teaching and research. It demonstrates how universities can improve the way they incorporate research into teaching to ensure that students can apply the knowledge they have gained in a practical setting. Pezaro et al. (2022) define research inspired teaching (RIT) as:

the integration of research into all teaching, learning and assessment activities and content as part of an authentic, disruptive and evidence-based inquiry that engages and inspires students and educators to think critically and to co-create innovative new knowledge, insights, transferable skills and impact. (p. 3)

**Figure 1.**
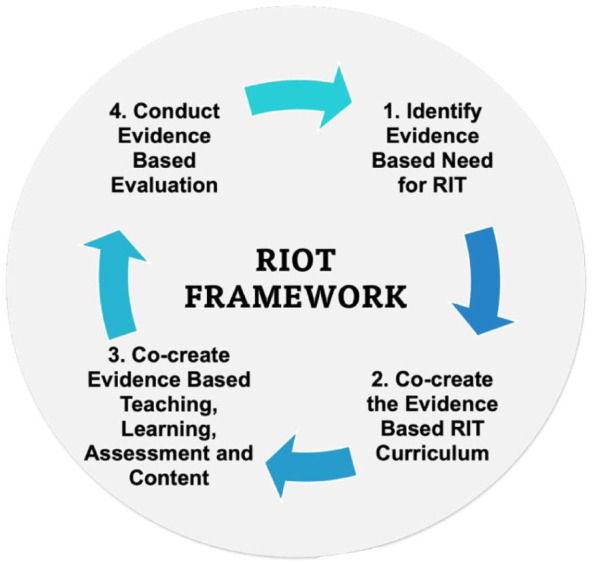
The RIOT framework. *Source*. Pezaro et al. (2022).

The RIOT framework provides a structured, cyclical approach to implementing RIT through four interrelated phases (Figure 1). In the first phase, the evidence-based need for RIT is identified by clearly defining and communicating this need, drawing on internal or external research or evaluative feedback that highlights knowledge gaps. The second phase involves the co-creation of the RIT curriculum, with stakeholders contributing diverse evidence bases, perspectives and expertise to collaboratively design a relevant and effective programme. The third phase focuses on the co-creation of evidence-based teaching methods, learning activities, assessments and content, with students actively involved in shaping their learning and formative assessment experiences. The final phase involves evidence-based evaluation to assess the effectiveness and replicability of the RIT initiative. The cycle then begins again, supporting continuous improvement of the curriculum and adaptation to emerging evidence-based and educational needs.

Before testing the applicability and feasibility of the RIOT framework, it is important to examine factors that may have an impact on its implementation. The aim of this research was to investigate the factors that influence the implementation of the RIOT framework into the curriculum and teaching practices, as well as finding solutions for overcoming identified barriers. The term “barrier” refers to any obstacle or hindrance that prevents a goal or action, including factors that limit or delay the adoption, implementation, or effectiveness of evidence-based practices (EBPs) and interventions (Nilsen, 2015; Powell et al., 2015; Proctor et al., 2013). In contrast, the term “overcoming” has been employed across a range of disciplines to signify an individual’s capacity to navigate, overcome, or surmount an issue, problem, obstacle or situation (Brush et al., 2011). By analysing the specific challenges that may limit knowledge acquisition and/or hinder change implementation, and the factors that may increase the likelihood of success in a particular context, strategies can be developed to ensure successful implementation (Graham et al., 2006). It is therefore crucial to identify and address barriers and find solutions to facilitate the advancement of RIT. Our research questions included: (1) What are the potential barriers that may affect the integration of the RIOT framework into curriculum and teaching practices in higher education? And (2) What approaches can be taken to mitigate the identified barriers?

## Methods

### Design and Setting

This study used a qualitative descriptive approach. Qualitative description, widely used in nursing research, is ideal for addressing real-world issues in a practical and accessible way (Bradshaw et al., 2017). Grounded in relativism and subjectivism, the approach is consistent with the study’s focus on understanding context-specific perspectives, where reality is seen as subjective and knowledge is co-constructed by researchers and participants (Bradshaw et al., 2017). The study was conducted at a United Kingdom (UK) based university, where academic roles tend to fall into one of two categories: teaching-focused or research-focused. Teaching staff are primarily responsible for teaching, learning, and assessment across a range of programmes, including pre-registration undergraduate degrees and postgraduate master’s-level education. These roles typically do not include a formal requirement to conduct research. In contrast, research staff principally engage in externally funded research, the supervision of post-graduate research students and producing research outputs with demonstrable academic and/or societal impact. There is little overlap in responsibilities between these two roles.

### Participants and Recruitment Process

Participants were recruited through purposive sampling, a method used to select individuals based on specific characteristics relevant to the study (Palinkas et al., 2015), combined with snowball sampling, where initial participants help identify additional candidates by referring others within their networks (Noy, 2008). Teaching staff were recruited via senior course leaders, while leadership and management staff were recruited via senior research and teaching leaders. Midwifery and nursing students were also invited to participate through student union representatives, who forwarded recruitment details to their cohorts. Approximately 110 members of teaching staff were invited to participate via an email, which provided information about the study. Email invitations were also sent to a cohort of healthcare educators, researchers and managers with an interest in RIT. Those interested in participating responded to the email, and an interview was arranged. An online survey was sent to those who were interested in participating but unable to attend an interview. Additionally, snowball sampling was employed, whereby participants were asked if they knew of other staff who might be interested in participating. A brief presentation of the study was also delivered at a monthly faculty meeting where participants were encouraged to contact the research team to schedule an interview or complete the survey form.

### Data Collection

Data were collected through semi-structured interviews and a qualitative survey between February and June 2024. While the concept of data saturation has traditionally been used in qualitative research, it is increasingly being questioned, particularly in applied fields such as education and health, where the diversity of experiences makes the idea of a clear saturation point problematic (Braun & Clarke, 2021; Saunders et al., 2018; Thorne, 2020). Considering the above, our focus remained instead on ensuring that there was a sufficient diversity of perspectives within the recruited sample to effectively address the aims of the study. This approach is consistent with the view that qualitative research should aim for a comprehensive understanding, based on the richness and diversity of the data, rather than a fixed notion of saturation (Thorne, 2016).

Participants were invited to take part in an interview, either in person or online via Microsoft Teams. Participants who were interested in taking part in the research but were unavailable for an interview had the option of completing an online survey via Jisc, a UK based digital platform supporting education and research. This ensured greater accessibility to participation. Both options covered equivalent content and invited either open text or verbal responses. All interviews were conducted by the same researcher (EL) and took an average of 30 to 60 min to complete. Participants were prepared for the interview by receiving background information about the project and were given further opportunity to ask questions about the project before the interview began. If the participants had not read the background information that was sent to them beforehand, a brief presentation of the RIOT Framework and its main features was provided prior to the commencement of the interview. The questions posed both in the interviews as well as the survey were: (1) What are the potential barriers to integrating the RIOT framework into the curriculum and teaching practice in higher education? (2) How can the barriers identified in research question one be overcome?

### Data Analysis

**Figure 2.**
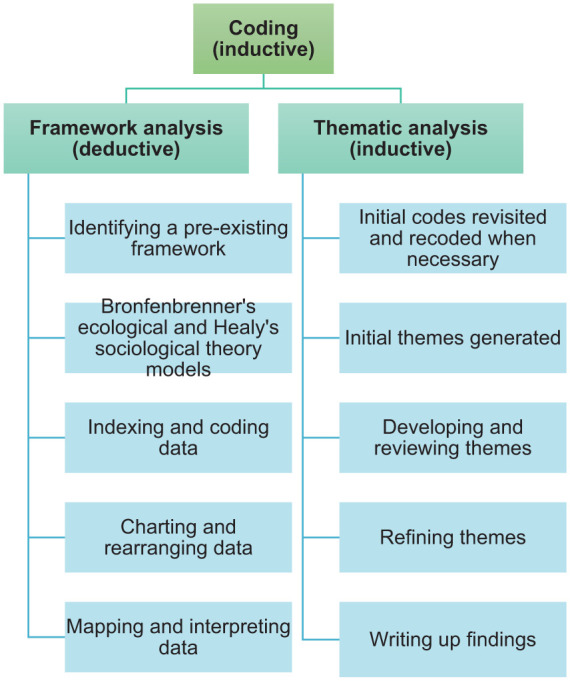
Flow chart of data analysis methods.

Interviews were audio recorded and transcribed verbatim. Any identifying information was removed from the transcripts prior to analysis. Data from both the interviews and surveys were extracted and analysed in the same way using Microsoft Excel and Microsoft Word. Due to the complexity of the data, the data was analysed at two levels, both deductively using framework analysis and inductively using thematic analysis. Figure 2 shows the flow chart of how the data were analysed using these two methods.

#### The First Layer of Analysis – Framework Method

The first stage of analysis involved familiarisation with the data and development of preliminary codes inductively. After initial coding, the first layer of analysis was completed using a deductive framework method of analysis (Ritchie & Lewis, 2003). A procedure for analysis was developed based on the steps introduced by Gale et al. (2013). A pre-existing framework to guide the analysis was identified. Two complementary frameworks were chosen for this study: Bronfenbrenner (2005) ecological model and Healy (2022) sociological theory model. A combination of the two frameworks was used, selectively integrating aspects of each to enhance their complementary effects. Both use a multi-level approach, dividing levels into micros, mesos and macros indicating that the process of human development is situated within a complex environmental context. In this study, individual levels explore the details of specific roles, tasks or immediate working environments, focusing on the day-to-day activities and responsibilities of individuals, in this case university staff. The interpersonal level examines the relationships and interactions between individuals, such as communication, teamwork, and collaboration. The organisational level refers to the department or faculty, including collaboration, organisational structure, processes and curricula. The institutional level focuses on the overall context, the whole institution, policies, mission and core values, and issues of strategy and leadership. In addition, the data included references to wider societal issues that affect the ability to maintain teaching excellence. The wider social and economic environment was thus established as a separate level.

Codes were grouped into levels according to how the content was associated with them. The following stages included indexing and coding data as well as charting and rearranging the data. The final stage included mapping and interpreting the data.

#### The Second Layer of Analysis – Thematic Analysis

As the data from the first layer revealed complexity and interconnectedness, a second layer of analysis was carried out to simplify the underlying themes and provide clarity and a deeper understanding of the topic. This second layer was completed using inductive thematic analysis as outlined by Braun and Clarke (2022). Thus, analysis began with familiarisation with the data, which had already been completed in the first layer of analysis. The initial codes established in this first level were then reviewed and recoded where necessary. From these codes, initial themes were generated. The subsequent step involved developing and reviewing these themes to ensure consistency with the codes and the overall data set. Once the themes were established, they were refined, defined, and named to clarify the overall narrative of the analysis. In the final stage, the findings were documented. The subjective and interpretive nature of data analysis was acknowledged throughout.

#### Member Checking

Following data analysis, findings were shared with research participants, who were invited to provide feedback and insights on them. They also had the opportunity to confirm whether they accurately reflected their experiences. Any omissions or discrepancies were raised and addressed during this process.

### Ethical Considerations

The study was granted ethical approval by Coventry University’s Research Ethics Committee (P168728). Prior to data collection, participants were given information about the study and written informed consent was obtained. Participation in the study was entirely voluntary, and all participants were free to withdraw at any point without consequence. Anonymisation was implemented through removing personal identifiers and assigning a unique participant number to each participant to protect their identity. These are used throughout the reporting process, instead of participant names or other identifying information. To further protect the anonymity of participants within a small and identifiable sample, data on sex and gender were not collected. This was considered particularly important given the potentially sensitive nature of the issues being explored within one faculty in a single educational institution. Moreover, as both midwifery and nursing are professions which predominantly consist of women, we considered that any men participating would be more easily identified should we have collected and reported such data in this context.

### Trustworthiness

The rigor of this study was supported by applying the criteria of credibility, transferability, dependability, and confirmability (Lincoln et al., 1985). To enhance the credibility of our study, methodological triangulation was employed, participants were given ample time to share their experiences, and member checking was conducted, allowing participants to review and confirm the findings. Transferability was supported by providing a description of the research context, participant characteristics and data collection procedures, which helps the reader to assess the applicability of the findings to other settings. Dependability was addressed by maintaining a detailed audit trail of the research process, including documenting the decisions made throughout data collection and analysis. The confirmability of the research was ensured through peer debriefing, where the findings were shared with the research team, as well as through keeping a reflexive journal.

### Positionality

The lead researcher is a PhD student at a UK university and a registered nurse and midwife from Finland. The research team included four other people, one from a midwifery, education and research background, one from a nursing, research and education background, and two from a non-health research and education role. The team recognised that since we all worked within the same university as the participants, this had the potential to influence data collection, our analysis, interpretations and role in reporting. To mitigate this, we engaged in reflexive journalling, and regular debriefing sessions with colleagues external to the research team. Here we were able to explicitly acknowledge and challenge any potential biases and assumptions. We were also aware of the power dynamic inherent with the researcher role. To mitigate against this, we prioritised: building rapport and trust with participants through transparent communication about the research purpose; ensuring voluntary participation and the ability to withdraw at any time; employing open-ended questioning techniques to encourage their perspectives; and actively seeking their feedback on our interpretations, to ensure their views and experiences were accurately represented. Lastly, we recognised that as both midwifery and nursing are professions largely dominated by women it would be important to counter potential biases with perspectives from the two members of the research team who were neither women, midwives nor nurses. As an all-white team, we lacked racial diversity. Whilst we did not collect data in relation to the race or ethnicity of participants, we acknowledge this as a significant limitation that may have influenced our perspectives, interpretations, and the questions we asked (or didn’t ask) throughout the research process. Importantly, none of the team members held student-facing roles, had responsibility for marking or moderating assessments, or were reporting managers for any participants. Additionally, all team members were based in different units from the teaching staff, which helped to reduce potential power imbalances related to institutional hierarchies.

## Results

A total of 30 participants took part in this study, with twenty-four interviews and six surveys submitted for analysis. The findings presented are derived from both the interview and survey data collectively. Participants were educators, researchers, leadership and management staff in the university. No students volunteered to participate. Table 1 shows the roles of participants.

**Table 1.** Roles of the Participants.

Role within the university
Senior leadership and management roles (n=5) For example, *senior academic leader, research and education leader, student experience and engagement leader*
Curriculum and academic development roles (n=5) For example, *academic developers, senior course leaders*
Teaching roles (n=9) For example, *lecturers, senior lecturers*
Research roles (n=11) For example, *assistant professors, professors*

### Findings from First Layer of Analysis

**Figure 3.**
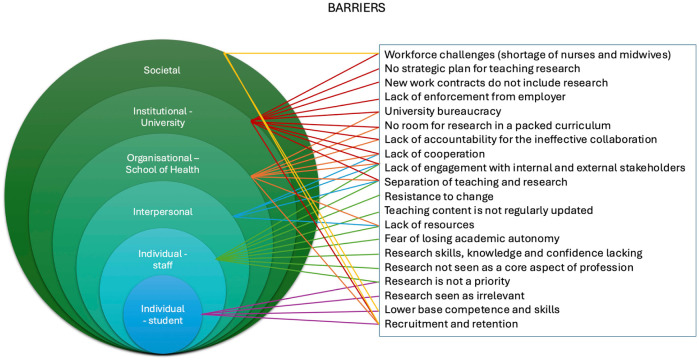
Illustration of barriers, highlighting the interrelationships with different contexts.

**Figure 4.**
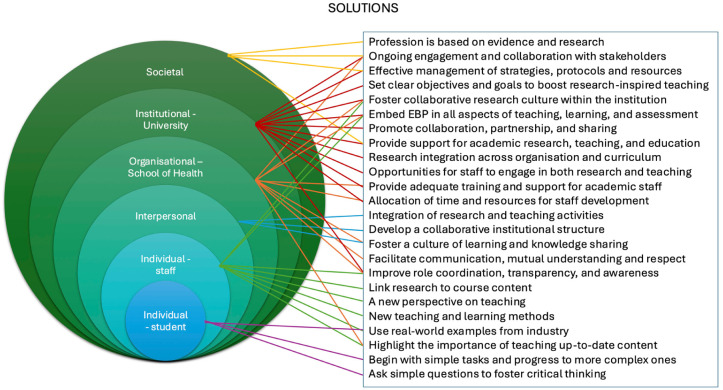
Illustration of solutions, highlighting the interrelationships with different contexts.

Barriers and solutions to integrating the RIOT framework were analysed across six levels: student, staff, interpersonal, organisational, institutional, and societal. A brief overview of each level is provided, followed by a description of how some of the barriers and solutions are interconnected. Figures 3 and 4 illustrate the links between the barriers identified and the solutions at different levels. The subsequent second-layer analysis presents a more detailed presentation of the barriers and proposed solutions identified during the first-layer analysis. Nevertheless, we briefly describe these below.

At student level, barriers included recruitment and retention challenges, limited research skills and scepticism about the relevance of research to clinical practice. Suggested solutions included making research more accessible and relevant by carrying out small-scale, evidence-based projects and structured critical thinking exercises, and improving communication about the future of the profession. At staff level, although some participants successfully integrated research into their teaching practice, significant challenges were highlighted in incorporating the framework, for example, the profession’s practice-oriented approach and traditional mindset, as well as the lack of recognition regarding the importance of research for professional practice. At the interpersonal level, weak collaboration and communication between educators and researchers were seen as key barriers to implementing the RIOT framework. Strengthening partnerships, aligning goals and developing communities of practice were identified as ways to promote a more integrated, collaborative approach to teaching. At the organisational level, siloed structures and the separation of teaching and research roles limited the integration of RIT. Participants called for more cohesive role design, integrated processes and flexible curricula to support research-informed education. At the institutional level, the implementation of the RIOT framework was constrained by policies, limited resources, rigid employment conditions and weak enforcement of research engagement. Suggested solutions included the strategic alignment of policies, improved resource allocation, and greater stakeholder engagement to prioritise research within education. Finally, at the societal level, broader challenges such as workforce shortages, limited research education at pre-entry level and financial pressures created systemic barriers to integrating the RIOT framework. Participants emphasised the importance of planning at a national level and stronger alignment between higher education and healthcare workforce strategies.

Several barriers and solutions were linked to multiple levels simultaneously rather than being confined to just one level. For instance, the separation of teaching and research was recognised as a single barrier with ramifications at institutional, organisational, interpersonal and individual staff levels. At an institutional level, this distinction is evident in the policies and organizational frameworks that delineate teaching and research as separate responsibilities. Organisational factors that reinforce this separation include siloed departments and fragmented roles. Interpersonally, this limits opportunities for collaboration between educators and researchers. At an individual staff level, this affects workload balance and the ability to integrate research into teaching practice. These overlaps highlight the complex, layered nature of the barriers and suggests that addressing them effectively requires coordinated action at all levels. Similarly, proposed solutions often required action or alignment across several levels to be effective, reinforcing the need for a coordinated, multi-level approach to implementation.

### Findings from Second Layer of Analysis

The second layer of data analysis yielded four core themes pertaining to the barriers and potential solutions associated with the integration of a RIOT framework into the curriculum and pedagogical practices: (1) understanding the relevance of research, (2) knowledge, skills, tools and confidence to apply research into teaching, (3) establishing a collaborative research culture and (4) overcoming challenges impacting the ability to maintain teaching excellence. Table 2 provides a summary of the themes, along with an overview of the barriers and solutions associated with each.

**Table 2.** Barriers and Solutions to Implementing the RIOT Framework in Midwifery and Nursing Education.

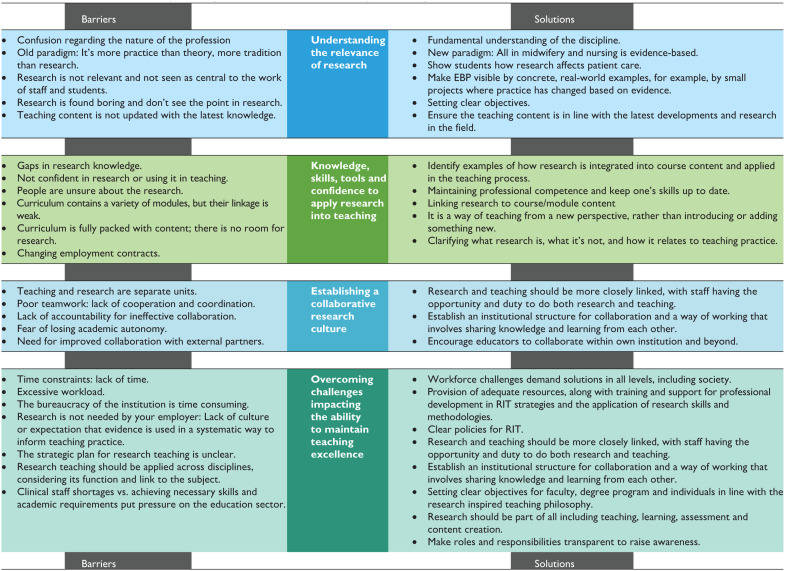

#### Understanding the Relevance of Research

The first theme is informed by two key findings in relation to the integration of the RIOT framework. First, participants reported that there appeared to be a lack of awareness of the importance of research among some students and faculty. Secondly, there was a perception that research was not seen as a core element of professional practice in nursing and midwifery. Participants expressed that many students enter the field with the expectation of focusing solely on practical and clinical work and that this might explain the poor commitment to research studies and general lack of interest, as illustrated below:

One of the issues also in nursing and midwifery is that a lot of people, students, when they come to learn that profession, they don’t think that research is part of it. They just go there to do the practical work, the clinical work. (P17, Research role)

Overall, research was generally viewed as dry and uninteresting, and according to some participants, students and staff often could not see the relevance of it. However, it was felt that academic staff working with future midwives and nurses have the potential to change this perception. It was suggested that this could be achieved by clearly explaining the subject and building confidence, particularly among those who may have a mixed understanding of the discipline. This could inspire students and ultimately encourage positive behavioural change in terms of how research is valued and applied.

Some educators saw research as an extra task. For example, as one participant describes, “It’s seen as an extra thing and not actually central to what they do, and I think that’s quite a major barrier.” (P10, Teaching role). Furthermore, it was noted that the already comprehensive curriculum posed challenges to incorporating more research into the curriculum. However, several participants asserted that implementing the framework is not merely about introducing new content into the curriculum, but rather about teaching from a renewed perspective. “We don’t need the students to become researchers . . . but we need them to become users of research and use their critical thinking skills and critical appraisal and understand how research is done” (P13, Leadership role). Another participant noted:

The vast majority of nurses, midwives, health practitioners that we educate, will become practitioners and stay at that level. And that is absolutely fine and fantastic, not everyone’s going to be a PhD researcher. But their mindset and their practice and their criticality within that role needs to reflect the fact that evidence-based practice is a cornerstone of what they should be doing. And there’s a risk that as finance gets tighter, we’ve just become a skills factory and we don’t invest enough in that side of it. (P22, Curriculum Development role)

A significant paradigm shift is occurring in midwifery and nursing, moving from practice dictated by tradition (e.g., “This is the way we do it because we have always done it”) to one centred on evidence-informed practice. This involves critically integrating research findings with clinical expertise and patient values to inform optimal decisions. However, there is variation among professionals in the field, as one participant indicated: “There’s people who are just doing what they were taught to do 50 years ago, and then there’s people who actually are, sort of, up with the latest thing, wanting to move things forward” (P9, Teaching role).

A much-discussed topic was the challenge of professionals not updating their teaching content. This meant that content could be outdated or at least did not include the latest evidence from the field. Preparing students for future roles means recognising the importance of research, staying current with content, and maintaining professional skills. Clear objectives and formal assessments were suggested to address these challenges.

#### Knowledge, Skills, Tools and Confidence to Apply Research into Teaching

The second theme describes the association between perceived RIT competence and the extent to which the RIOT framework is integrated. The primary obstacles encountered in this category were a lack of comprehension of research, an inability to apply it to practice, and a shortage of time. Some participants expressed lack of staff confidence in linking course content with research or finding relevant information. Participants reflected how midwifery and nursing have a less extensive research history than other fields. Not all those engaged in educational activities have undergone training in research methodology, nor do they necessarily possess a research background. Given these barriers, it was suggested that better organisational support should be offered for staff along with collaboration with internal and external partners:

Sometimes it may be upskilling, sometimes it may need to be collaborations and working along with projects. Academics and clinicians joining hands together and doing, and sharing their knowledge as well will help with this I would suggest, and we have very good networks within university with our local partners. (P19, Teaching role)

One important aspect brought to discussion were the presence of ever changing employment contracts. Previously, academic contracts for lecturers or assistant professors included an expectation of research and/or scholarly activities. More recently, new hires as teaching fellows or senior teaching fellows have different contracts, with no obligation to participate in research. Participants viewed this approach as a shift in thinking, emphasising that educators shouldn’t be expected to conduct research unless they choose to. Instead, the focus should be on ensuring that everyone in the field uses it. One participant noted:

The majority of people, you know, find research scary. We need to upskill staff in the school . . . Some people from practice I think have preferred the fact that it’s a teaching fellow, and that it doesn’t have research associated with it because research is something they’re scared of . . . When I’ve done teaching observations, you know, people use guidelines rather than using empirical research. I know it’s better than nothing . . . So, I think currently it’s very superficial. (P1, Curriculum development role)

In order to promote staff confidence, participants advised that illustrative examples drawn from both internal and external sources be utilised to demonstrate the effective incorporation of research into teaching content. Staff also indicated a need for support to develop their research knowledge, skills and confidence, and a recommendation was made for a systematic process to be implemented within the institution to meet these needs. Furthermore, it was considered vital to engage all relevant parties and stakeholders in the collaborative development of solutions for teaching practice.

#### Establishing a Collaborative Research Culture

The third theme captured aspects of creating, recognising and developing collaborative research environments. An issue that emerged in almost all interviews was the organisational division of research and teaching and the challenges that this posed to implementation of the RIOT framework. The importance of collaboration and co-creation was highlighted as a key strategy for supporting the integration of research and the RIOT framework. The current organisational strategy aims to enable researchers to focus solely on research and educators on teaching. Participants questioned whether educators should be enabled to conduct their own research and debated the merit of research being the prime responsibility of research centre staff, who are located within units external to the faculty. It was considered that the connections between research centres, education and clinical practice posed a challenge, creating further boundaries. The problems identified linked to accountability, co-operation, communication, mutual respect and clarity of roles within the organisation. Participants emphasised the need for transparency, openness and awareness of what everybody does and what everybody’s role is in this task.

The solutions emphasised the importance of cultivating robust partnerships, facilitating open communication, and establishing shared objectives. Participants proposed that communities of practice comprising educators and researchers could play a pivotal role in facilitating the implementation of the RIOT framework. Some participants suggested that it might be beneficial to explore the introduction of additional tools and materials, organised in a way that seamlessly integrated teaching and research, allowing both researchers and educators to benefit from each other’s expertise. To facilitate change, one participant suggested that collective accountability should be taken by all stakeholders. Another participant suggested:

Maybe joint ventures or opportunities for the teaching staff to learn a little bit more about what you’re doing, and you know where anybody is, physical things who, who is who, maybe some of us being involved in the research that people are doing. Just more co-working really. (P12, Teaching role)

From the perspective of the RIOT framework, it was considered imperative to strengthen the link between research and teaching, ensuring that staff have both the opportunity and the obligation to engage in both activities. Establishing an institutional structure to support collaboration was viewed as essential, along with developing a collaborative working approach that fosters knowledge sharing and mutual learning. The aim of these activities is to motivate educators to collaborate within their institutions and to foster relationships with educators outside their institutions. Collaboration with health care institutions was also suggested as a way of integrating real-world examples into educational programmes and to better align the integration of research with practice. In addition, the importance of maintaining collaborative relationships was recognised, given the ongoing need for dialogue with external stakeholders on best practice.

#### Overcoming Challenges Impacting the Ability to Maintain Teaching Excellence

The final theme concentrated on factors that have a significant impact on the effective maintenance of teaching excellence and integration of RIT. The limitations of available resources and time, coupled with the heavy workloads faced by staff, constitute a significant challenge for integrating research into teaching practice. It was acknowledged that people have a sense that their schedules and those of their colleagues are full and that they are overwhelmed by the demands of the day-to-day duties. Many found it unrealistic to remain up to date with the latest evidence-based research or to find time for collaborative activities. These challenges were intensifying due to a shortage of staff and mounting pressures, as the excerpt below exemplifies:

At a policy level, I can see that it’s all like forward thinking, but on a practical level, many departments, including mine, are quite understaffed and so it’s practically not possible. It gets pushed back towards the long list of things to do, so it’s practically - there’s not enough time for staff to even deliver their day-to-day duties. (P19, Teaching role)

For the overall institutional aim of ensuring that research is effectively integrated into teaching and learning, participants expected a clearer strategy and protocols for implementing the RIOT framework, and support for the application of research to teaching practice. Participants emphasised that, if a university wants to promote RIT in the organisation, research should be embedded in all teaching and learning activities (teaching, learning, assessment, and content). In that way research would be integral part of teaching activity and be in line with the research-informed teaching philosophy. It was mentioned that:

That needs to be an organisational priority. It should be in everybody’s development plan, and it should be in a departmental agenda as well, like how are we developing ourselves, how are we contributing to this framework, how can we implement this and what to do . . . There is a gap between clinicians and academics, and what we teach and what research we expect, like the positive disruption and bridging the gap between books and practice. That needs to be challenged as well. (P21, Leadership role)

As a solution, setting specific and clear objectives for faculty, degree programmes and individuals according to the desired outcome was offered. Moreover, well-designed strategies, protocols, and resources that promote research inspired teaching to guide daily activities, along with clear role coordination (goals, roles, and responsibilities for each team), and encouragement were suggested. Participants emphasised the need for transparency, openness and awareness of what everybody does and what everybody’s role is in this task.

One of the significant challenges in maintaining teaching excellence in midwifery and nursing is the shortage of qualified educators. With the increasing demand for healthcare professionals, there is a constant need for experienced healthcare practitioners to transition into teaching roles. One participant summed this up – “The higher education landscape for nursing is quite challenging at the moment. We need more higher education staff . . . And for us recruiting at the minute is a real challenge because there simply just isn’t enough nurses” (P16, Research role). Educators felt that they were expected to undertake an increased workload, despite already being understaffed. The situation created stressful experiences, impacting the overall quality of education.

### Model Development: Synthesizing Insights from Analysis

**Figure 5.**
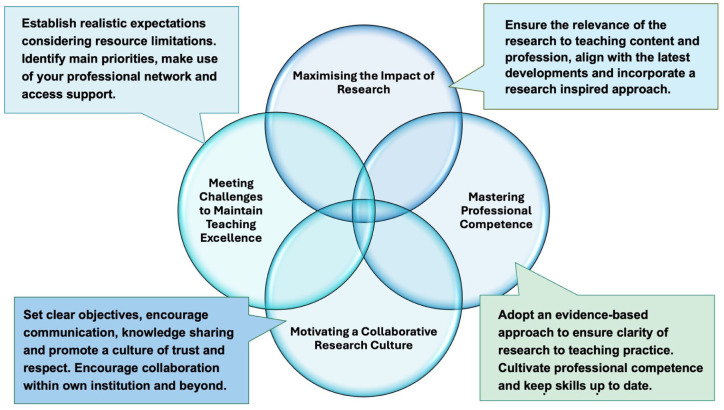
The 4M Model for promoting research inspired teaching in midwifery and nursing education.

In pursuit of model development, the findings from both the framework and thematic analyses were synthesised by comparing and combining similar ideas across predefined categories and emergent themes, while also noting key differences. This process led to the development of a conceptual model titled *the 4M Model for Promoting Research Inspired Teaching in Midwifery and Nursing Education* (Figure 5). This model provides a clear, concise and transferable summary of the findings and serves as a tool to support the implementation of the RIOT framework in healthcare settings and educational practice across the sector.

The following key factors were identified as being essential for promoting RIT and supporting the successful implementation of the RIOT framework:

(1) *Maximising the impact of research* means clarifying the relevance of research and gaining a comprehensive grasp of the discipline’s fundamentals and how research and EBP relate to professional practice. It also involves aligning the content of teaching with the latest developments and incorporating a research inspired approach part of all work.
(2) *Mastering professional competence* entails mastering research and EBP knowledge and skills, as well as keeping one’s skills up to date. In practice, this means identifying and implementing examples of research inspired content and teaching strategies in the design and delivery of educational programmes. Moreover, it is encouraged to provide resources, training, and ongoing support for professional development in RIT and research application skills and methodologies.
(3) *Motivating a collaborative research culture* includes encouraging educators to collaborate within their own institution and beyond. It means forming an institutional collaborative structure and setting up a clear way of working together, sharing knowledge, communicating and learning from each other.
(4) *Meeting challenges to maintain teaching excellence* refers to the ability to overcome complex challenges and maintaining the highest standards of teaching despite the difficulties that may be encountered.

## Discussion

The findings of this study provide valuable insights into the factors influencing the integration of the RIOT framework into the curriculum and teaching practice in higher education programs for midwifery and nursing. The integration of the Bronfenbrenner’s ecological model and Healy’s sociological theory model in the framework analysis, combined with thematic analysis was instrumental in situating individual experiences within the broader systemic contexts. This enhanced understanding of societal trends, interrelationships, and factors influencing midwifery and nursing education. Implementing the RIOT framework involves more than introducing a course module or teaching approach; it also requires examining key organisational aspects and systemic changes influenced by societal trends. This analysis allowed us to consider a broader range of factors related to the implementation of the RIOT framework, including the conditions necessary to support policies and programmes that promote it. It also shifted the focus from individual responsibility to a broader understanding of the social and environmental factors (see Ewald et al., 2023) that influence the work of professionals. Incorporating these two levels of analysis was crucial in developing the 4M model, which provides a basis for guiding the integration of research at both the individual and collective levels in midwifery and nursing education, guides the implementation of the RIOT framework, and holds potential for wider application.

The process of transferring research findings into healthcare in educational or clinical settings is often impeded by a deficiency in knowledge, expertise, and confidence among healthcare professionals (such as midwives, nurses, and educators) in utilising evidence-based sources, such as research outcomes, in their practice (Bäck et al., 2017; Clarke et al., 2021; Dagne & Beshah, 2021; Duncombe, 2018; Melnyk & Raderstorf, 2019; Saunders & Vehviläinen-Julkunen, 2016). Limited time, resources and high workloads further hinder EBP and innovative teaching strategies (Dagne & Beshah, 2021; Horntvedt et al., 2018; Melnyk & Raderstorf, 2019; Saunders & Vehviläinen-Julkunen, 2016). Although research is becoming increasingly important across fields, our findings reveal that some students and educators view it as neither relevant nor central to their work, leading to low engagement. Participants also noted mixed perceptions of these professions, with many seeing midwifery and nursing as more practice-oriented than research-based.

To integrate the RIOT framework, participants suggested the need for pragmatic, practice-based approaches to teaching that provide tangible, relevant learning opportunities for students. Concrete examples are needed to facilitate the integration of research into course content and teaching methods, as well as the application of research findings to patient care and knowledge of best practice. This was also one of the main conclusions of a recent systematic literature review on this topic (Leiviska et al., 2025). In response to these needs, the 4M model was developed as a tool to help educators implement the RIOT framework. The aim of the RIOT framework is to ensure the practical relevance of RIT, to provide clear action plans and to equip teachers and working communities with the necessary tools to achieve it. The overall aim of these efforts is to systematise the integration of research into the teaching process, thereby increasing its accessibility and effectiveness.

Difficulties in collaboration and dysfunctional group dynamics can adversely affect clinical education (Hakim, 2023), as can failure to foster a culture that values research and innovation (McNett et al., 2023). The main identified obstacles to implementing the RIOT framework in this study were the organisational separation between research and teaching departments and the lack of knowledge exchange between them. It was observed that the establishment of networks and collaborations within and across professional associations can enhance the efficacy of knowledge and resource sharing, thus facilitating the dissemination of research findings, best practices, and innovative pedagogical approaches. Prior research has highlighted barriers to research implementation, including inadequate support structures, limited training opportunities, poor information dissemination, lack of incentives and leadership, and poor evidence synthesis (Mathieson et al., 2019; Melnyk & Raderstorf, 2019). Similarly, our study participants emphasised the need for training and support to implement the RIOT framework, highlighting the impact of organisational constraints.

The present study also identified two novel barriers yet to be discussed in the literature in this context. The first barrier is the declining knowledge base in basic skills, such as literacy, among students entering higher education. The second barrier is the global shortage of midwives and nurses in both educational and clinical contexts. It is crucial to address these challenges with targeted initiatives that build fundamental abilities in incoming students and attract and retain skilled professionals in education and healthcare. This dual approach can help ensure the continued excellence and sustainability of midwifery and nursing education that is research inspired.

Healthcare degrees are a higher education qualification offered by universities in partnership with clinical placement providers. The successful management of university-level studies requires a range of academic and critical thinking skills. While some participants in our study have raised concerns about students’ preparedness, especially regarding literacy and scientific reasoning, this issue should be understood within a broader educational context. As Wingate (2015) argues, student diversity in higher education necessitates inclusive academic literacy practices, and in-sessional support initiatives have been implemented to assist students in developing the necessary competencies. This is particularly relevant as participation in disciplines such as nursing, midwifery, and other health-related programs continues to widen.

Concerns about basic skill levels remain, with a recent global study (Gust et al., 2024) reporting that 63% of secondary school students worldwide fail to meet Programme for International Student Assessment (PISA) Level 1 skills (e.g., the abilities needed to participate effectively in the modern international economy). Nevertheless, rather than interpreting this solely as a decline in ability, it is important to consider the role of pedagogy and support in this context. Boshnjaku et al. (2023), for example, identify a lack of scientific knowledge and critical thinking as potential barriers to the development of EBP competencies, but they also emphasise that a variety of educational strategies have proven effective in improving student outcomes. Similarly, a systematic review and meta-analysis by Jeong et al. (2024) reports that EBP education programs can significantly enhance nursing students’ critical thinking, problem-solving, and EBP competencies. In our study, some staff members cited literacy challenges as a barrier to student engagement with research and EBP content. These perceptions underscore the importance of continued pedagogical innovation and support to ensure inclusive participation. Future research and initiatives should address both perceived and actual skill gaps while building on existing evidence of effective teaching strategies within HEIs.

Another issue is the global shortage of midwives and nurses (Nove et al., 2021; World Health Organization [WHO], 2020). Enhancing the capability for education and training represents an efficacious solution to meet prospective requirements for care. Indeed, the NHS Long Term Workforce Plan (NHS England, 2023) sets ambitious targets for increasing training places in all NHS clinical professions, including almost doubling the number of nursing places in England by 2031/2032. As part of the newly launched 10-Year Health Plan for England, there is also an emphasis on expanding professional development and improving access to high-quality training and education (Department of Health and Social Care, 2025). However, our findings demonstrate that educators are already experiencing challenges in providing quality education due to the heavy workload and high expectations of producing new professionals in the field with the necessary skills. The resolution of additional demands on educators necessitates the collaborative endeavours of healthcare organisations, educational institutions and policymakers to attract and retain proficient professionals, enhance the quality of resources, and prioritise the significance of pedagogical excellence within the healthcare domain (Evans et al., 2017). The dynamic nature of society and the evolving healthcare landscape influence the internal and external developments of higher education. Tackling the global shortage of midwives and nurses will require a collective effort to strengthen educational capacity and resources, and to ensure that educators can effectively produce skilled professionals while adapting to the changing demands of health care.

### Strengths and Limitations

A key strength of this study was the selection of participants with relevant characteristics. By interviewing a range of stakeholders, a variety of perspectives were captured, providing a deeper insight into educational challenges and opportunities. This multifaceted approach ensured that the findings reflect the complexities of integrating research into teaching practice. In addition, qualitative interviews provided in-depth insights into participants’ thoughts and feelings, offering a comprehensive understanding of complex issues while allowing flexibility to explore unexpected themes.

Although the participants came from diverse backgrounds, there remains the potential for selection bias, as individuals with an interest in the topic were more likely to engage in the interviews, while those lacking interest may have been less inclined to participate. Future studies could mitigate this by using broader recruitment strategies and/or mixed methods approaches to capture a wider range of insights. In addition, the lack of student participants limits the scope of the findings. Future research should include student perspectives to provide a more balanced understanding. The findings may not be generalisable to settings beyond HEIs that do not share similar roles, organisational structure, support, resources and academic philosophies.

### Implications to Midwifery and Nursing Education

Our research highlights several important teaching strategies to improve the integration of research into teaching and clinical practice via the RIOT framework. Key to this is a basic understanding of the discipline and an awareness that midwifery and nursing practice is evidence-based. It is important to show students how research has a direct impact on patient care by using real-life examples, such as both small projects or larger scale studies where practice has changed as a result of evidence. Strategies that could be used to mitigate doubtful attitudes and beliefs about research include starting with simple methods and progressing to more complex tasks and encouraging critical thinking through simple questions. Aligning teaching content with the latest developments in the field is crucial. Educators struggle with time constraints, competing priorities, and lack of confidence in their research skills. Encouraging educators to co-create, collaborate and maintain an ongoing dialogue within their institution and with external stakeholders on best practice could be a valuable method of finding solutions and examples to pedagogical challenges. Providing training and support for professional development in research application strategies and methodologies is key to achieving these goals.

### Implications for Research

The relationship between teaching and research is complex and requires further exploration to improve understanding and inform practice. Integrating teaching, research and practice could yield significant benefits. Identifying solutions to overcome barriers is essential for testing the feasibility and applicability of the RIOT framework and should be considered during its implementation. Identifying strategies to alleviate stressors faced by learners and educators is essential to foster a supportive environment for responsible practice.

## Conclusion

This comprehensive study identified the key elements influencing the implementation of the RIOT framework demonstrating it is not merely the application of a new approach to teaching. Rather, it is a multidimensional institutional and societal discourse about the future of midwifery and nursing. Overcoming the barriers in midwifery and nursing necessitates a collaborative effort to tackle several challenges, including the shortage of professionals, the demands of higher education, competency requirements, heavy workloads, and the need to enhance the effectiveness of teaching strategies. By addressing these challenges, all stakeholders will be better equipped with the skills and expertise needed to effectively develop and implement the RIOT framework in healthcare education. The 4M model provides a roadmap for how this may be achieved.
